# Using open-source intelligence to identify early signals of COVID-19 in Indonesia

**DOI:** 10.5365/wpsar.2020.11.2.010

**Published:** 2021-02-17

**Authors:** Yoser Thamtono, Aye Moa, Chandini Raina MacIntyre

**Affiliations:** a Biosecurity Program, Kirby Institute, Faculty of Medicine, University of New South Wales, Sydney, Australia.

## Abstract

**Objective:**

Open-source data from online news reports and informal sources may provide information about outbreaks before official notification. This study aims to evaluate the use of open-source data from the epidemic observatory, *EpiWATCH*, to identify the early signals of pneumonia of unknown cause as a proxy for COVID-19 in Indonesia.

**Methods:**

Using open-source data on pneumonia of unknown cause in Indonesia between 1 November 2019 and 31 March 2020 (extracted from *EpiWATCH*, an open-source epidemic observatory), a descriptive analysis was performed to identify the trend of pneumonia of unknown cause in Indonesia before official notification of COVID-19 cases.

**Results:**

A rise in reports of pneumonia of unknown cause was identified in Indonesia, starting from late January 2020. There were 304 reported cases of pneumonia of unknown cause, 30 of which occurred before the identification of the first COVID-19 cases on 2 March 2020. The early signals of pneumonia of unknown cause in Indonesia may indicate possible unrecognized circulation of severe acute respiratory syndrome coronavirus 2 (SARS-CoV-2) before official detection.

**Discussion:**

Open-source data may provide rapid, unvalidated information for early detection of outbreaks. Although unvalidated, such information may be used to supplement or trigger investigation and testing. As *EpiWATCH* sources global information, this methodology can be repeated for other countries within the Western Pacific Region, and for other diseases.

On 31 December 2019, China reported an increased occurrence of pneumonia of unknown cause in Wuhan, (*1*) which was later confirmed as coronavirus disease 2019 (COVID-19), a respiratory illness caused by the newly discovered coronavirus, severe acute respiratory syndrome coronavirus 2 (SARS-CoV-2). On 13 January 2020, Thailand became the first country to identify imported cases of COVID-19. Other countries in South-East Asia, including Singapore and Malaysia, also reported their first imported cases in January 2020. (*2*, *3*) On 31 January 2020, the World Health Organization (WHO) declared COVID-19 a public health emergency of international concern, (*4*) and on  11 March 2020, it declared the outbreak a pandemic. (*5*) Indonesia reported its first two confirmed positive cases of COVID-19 on 2 March 2020. (*6*)

Early identification of an infectious disease outbreak is essential, to allow for immediate initiation of public health interventions and to mitigate global impacts of transnational spread of the disease. Traditional public health surveillance, especially where there is low testing capability, is often not timely due to delays in reporting and validation by local health authorities. (*4*)

Open-source intelligence may serve as a valuable tool for rapid epidemic surveillance. (*5*) WHO reports that more than half of early epidemic information can be obtained through unofficial sources, including online news outlets and social media. (*6*) This study aims to evaluate the use of open-source data from the epidemic observatory, *EpiWATCH*, to identify the early signals of pneumonia of unknown cause as a proxy for SARS-CoV-2 circulation in Indonesia.

## Methods

*EpiWATCH* is a semi-automated open-source epidemic observatory that collects and analyses outbreak data from publicly available sources, such as online news outlets and social media. (*7*) *EpiWATCH* has been used to collect outbreak data since 2016. The observatory collects information on outbreaks of diseases and emerging infections, globally, to detect early signals and trends, which can then be used by researchers. The system was established and is managed by the Australian National Health and Medical Research Council (NHMRC) Centre for Research Excellence, Integrated Systems for Epidemic Response (ISER). (*7*)

*EpiWATCH* gathers open-source data from online news outlets and social media through an intelligent and modular system. To enable enhanced surveillance, searches are modified for specific languages and for specific infectious disease syndromes. Modifications include changing and adding keywords and languages for searching. The observatory system supports various intelligent data-gathering algorithms, including natural language processing algorithms, regular expression matching and supervised machine-learning algorithms. The data are stored in a PostgreSQL database. (*8*) Within *EpiWATCH*, reports are reviewed and entered manually by the team, to ensure collection of key data points identified by the automated data-gathering system and prevent duplication of reports.

In this study, enhanced surveillance was performed through *EpiWATCH* using keywords reflecting SARS or pneumonia, such as “pneumonia, lung infection, lung inflammation, severe acute respiratory infection (SARI), cough, fever, cough AND fever,” with Indonesia as a location, in Bahasa Indonesia (the country’s official language). The obtained reports were manually reviewed, and any reported cases of pneumonia of unknown cause in Indonesia dated between 1 November 2019 and  31 March 2020 were included in the *EpiWATCH* database. Reports matching the keywords were analysed and were excluded if they were not reporting cases from Indonesia or cases of pneumonia of unknown cause, or if they were reporting duplicate news or pneumonia cases with a known cause. Indonesia was chosen for this study as its first official COVID-19 notification was made later than that of other South-East Asian countries.

A descriptive epidemiological analysis was performed using Microsoft Excel 2016 to group cases by geolocation and by the date on which they were reported. Data reported from Indonesia’s Ministry of Health were also reviewed and compared with the news reports.

### Ethics and permissions

No ethics approval was required for this study because it uses publicly available open-source data from online media.

## Results

Between November 2019 and March 2020, *EpiWATCH* documented 217 entries or reports related to pneumonia of unknown cause in Indonesia. Six duplicates were removed, leaving a total of 211 included reports. The highest number of reports (184 entries) were from  March 2020, after the official identification of the first cases of COVID-19 in Indonesia. However, a steady increase in the reports of pneumonia of unknown cause was seen from late January 2020 (five entries) to the end of February 2020 (17 entries).

These 211 entries correspond to 304 reported cases of pneumonia of unknown cause in Indonesia, 30 (9.9%) of which occurred before the identification of the first COVID-19 cases on 2 March 2020. Among those 30 cases, 17 were suspected for COVID-19, including the case of a toddler from China, aged 18 months, who was treated for pneumonia in Lombok, West Nusa Tenggara, on 27 January 2020. (*9*) COVID-19 diagnosis was ruled out based on a blood laboratory count that was suggestive of bacterial infection; however, no further results of polymerase chain reaction (PCR) or bacterial culture were reported. (*9*)

**Figure 1:**
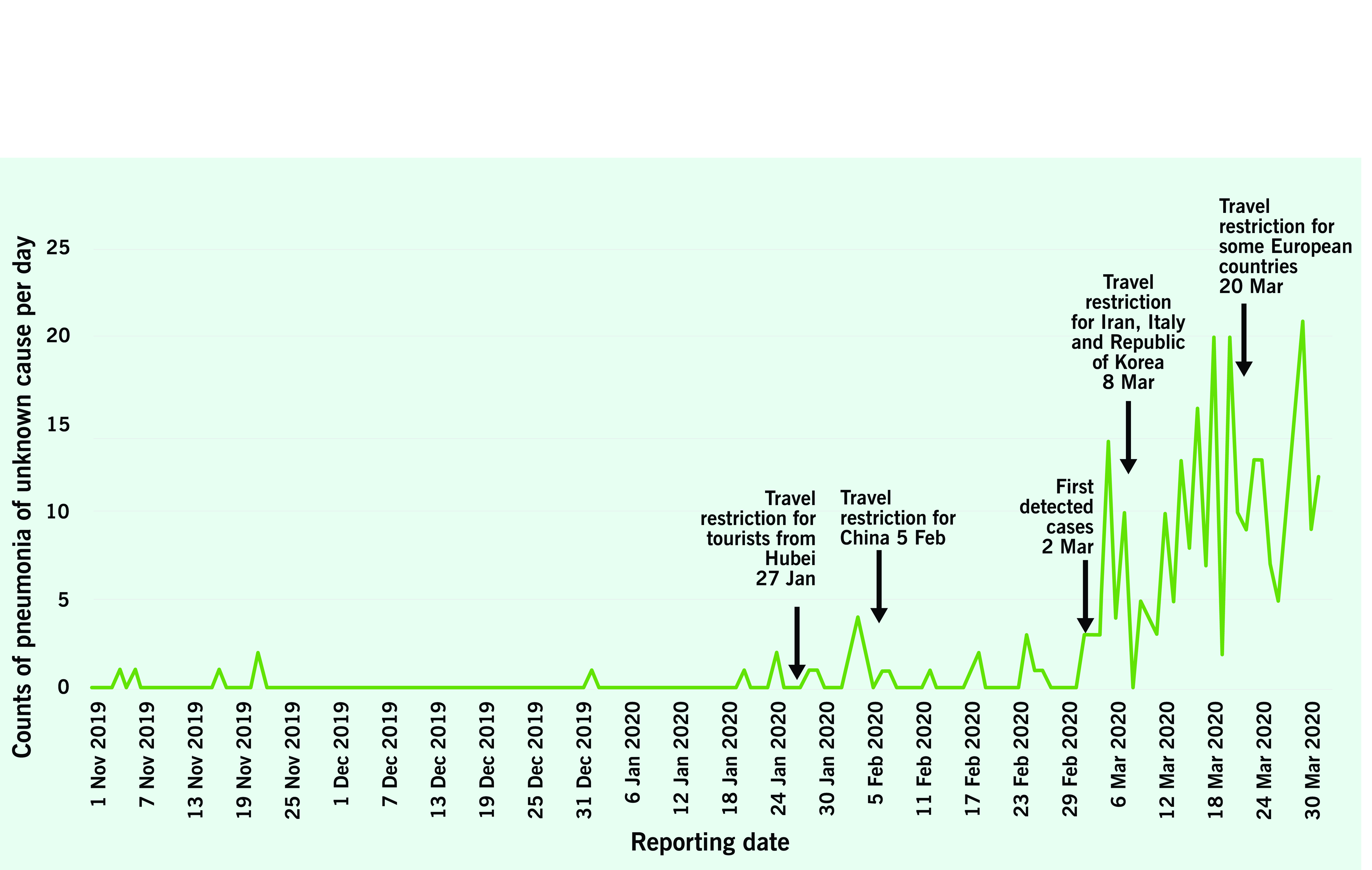
Number of EpiWATCH reported cases of pneumonia of unknown cause in Indonesia between 1 November 2019 and 31 March 2020

**Fig. 1** shows the count of pneumonia of unknown cause from 1 November 2019 to 31 March 2020 as obtained from *EpiWATCH* reports. In November and December 2019, there were five *EpiWATCH*-reported cases of pneumonia of unknown cause. The number of reports increased slightly from late January (six identified cases) to February 2020 (19 identified cases), six weeks before the official identification of the first two COVID-19 cases in Indonesia. In March 2020, the count of *EpiWATCH*-reported cases of pneumonia of unknown cause increased significantly to 274 cases, which may represent an increase in COVID-19 cases or growing media attention and reporting following the official notification of COVID-19 in Indonesia on 2 March 2020. In the same period in the previous year, there were far fewer reports of pneumonia of unknown cause, with only five *EpiWATCH*-reported cases of pneumonia of unknown cause from January to March 2019.

[**insert** Figure 1]

**Figure 2:**
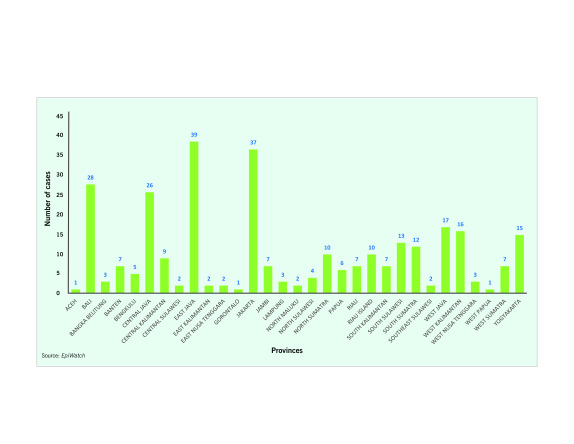
Number of EpiWATCH reported cases of pneumonia of unknown cause in Indonesia, by province (November 2019 to March 2020)

**Figure 3:**
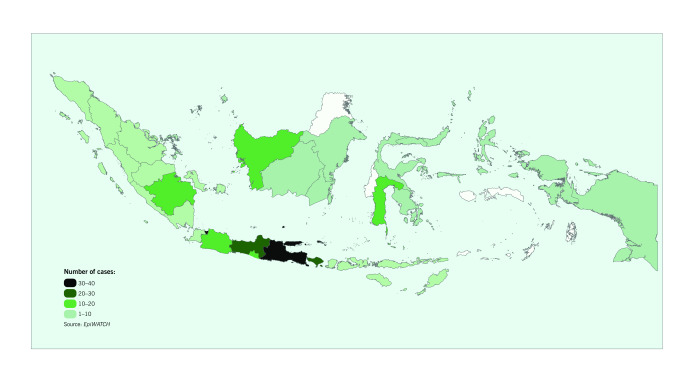
Geographical distribution of EpiWATCH reported cases of pneumonia of unknown cause in Indonesia (November 2019 to March 2020)

Almost half of all *EpiWATCH*-reported cases of pneumonia of unknown cause were from provinces in Java. East Java (39 cases; 12.8%) and Jakarta  (37 cases; 12.1%) were the provinces with the highest number of *EpiWATCH*-reported cases, followed by  Central Java (26 cases; 8.5%), West Java (17 cases; 5.6%), Yogyakarta (15 cases; 4.9%) and Banten (seven cases; 2.3%). Outside Java, Bali had the highest number of *EpiWATCH*-reported cases (28 cases; 9.2%) (**Fig. 2** and **Fig. 3**).

[**insert** Figure 2]

[**insert** Figure 3]

Gender information was given for 157 of the 304 *EpiWATCH*-reported cases. Among those 157 cases, there was a higher proportion of males (96 cases; 61.1%) than females (61 cases; 38.9%), with a 1.5:1 ratio of males to females. Information on age was given for 138 of the 304 *EpiWATCH*-reported cases, with the highest proportion of cases being those aged 50–60 years.

## Discussion

This study provides a descriptive analysis of cases of pneumonia of unknown cause that occurred in Indonesia from November 2019 to March 2020 as detected by the *EpiWATCH* observatory. These *EpiWATCH*-reported cases of pneumonia of unknown cause in Indonesia increased from late January 2020, which may reflect the presence of COVID-19 cases in the country before official identification of two cases at the start of March 2020.

Data from the Ministry of Health (*10*) show that, by  31 March 2020, Indonesia had 1528 laboratory- confirmed cases of COVID-19, with 136 documented deaths. Among these cases, almost half (48.9%) were identified in Jakarta, followed by other provinces in Java: West Java (11.9%), Banten (9.2%), East Java (5.9%), Central Java (5.2%) and Yogyakarta (1.2%). (*10*) At that time, Bali had reported only 19 confirmed cases of COVID-19 (1.2% of total cases). (*10*)

The spatial distribution of notified COVID-19 cases in Indonesia is similar to that of pneumonia of unknown cause from *EpiWATCH*, suggesting the potential use of pneumonia of unknown cause as a proxy for COVID-19 cases in Indonesia. However, there were proportionately more notified cases from Jakarta compared with the spatial distribution of pneumonia of unknown cause from *EpiWATCH* in our study. This might suggest geographical differences in the ability to identify and report COVID-19 cases. Health infrastructure gaps have been cited as one of the most critical issues in the Indonesian health system. (*11*) Health facilities, including hospital and laboratory services, are more readily available in the urban Java region, where Jakarta, the capital city, is located. (*12*)

Provinces in Java, especially Jakarta, might have better capability for identifying cases of COVID-19. Meanwhile, other provinces may have underdetection of COVID-19 cases; for example, Bali notified a low number of confirmed COVID-19 cases compared with the *EpiWATCH*-reported cases of pneumonia of unknown cause. Of the 12 COVID-19 national reference laboratories in Indonesia, only three are located outside Java, which may hinder the ability of provinces outside Java to rapidly identify and respond to the presence of COVID-19. (*13*)

This study is the first of its kind to describe the epidemiological pattern of pneumonia of unknown cause reported from open-source intelligence before and after the official notification of COVID-19 cases in Indonesia. It shows that monitoring trends of pneumonia of unknown cause in open-source data could provide rapid, unvalidated information for early detection of COVID-19 outbreaks. Although the information is unvalidated, once a signal is detected, it could prompt further investigation and validation. Consequently, such information may supplement traditional surveillance, which is frequently subject to delays in reporting and validation by local health authorities. (*4*)

This study has several limitations. First, there is a possibility of reporting bias because we may have captured increasing media awareness rather than an actual increase in disease occurrence. The early signal was detected in late January 2020, when COVID-19 had been detected in many countries worldwide. Second, this study relies on an online news-based surveillance system, which is unvalidated and may include other etiologies of pneumonia that were not COVID-19. (*14*) Third, we only included pneumonia in our study; however, COVID-19 has a wide range of symptoms, with most of those infected having a mild illness. (*15*) Nevertheless, this study has highlighted the ability of open-source data to identify early alerts of pneumonia before the initial confirmation of COVID-19 cases in Indonesia, making such data a promising option to enhance epidemic surveillance.

## Conclusion

In this study, we detected possible early signals of the COVID-19 outbreak in Indonesia using an online news-based surveillance system that used Bahasa Indonesia through *EpiWATCH*. The earliest signals from the *EpiWATCH* observatory of pneumonia of unknown cause in Indonesia were in late January 2020, indicating possible unrecognized circulation of COVID-19 cases in Indonesia before the country’s official notification of cases. The observed spatial pattern of cases between *EpiWATCH*-reported pneumonia of unknown cause and officially confirmed COVID-19 cases in Indonesia was similar.

Monitoring trends in open-source data can provide rapid, unvalidated information for the early detection of outbreaks. Although unvalidated, such information may be used to supplement or trigger investigation and testing. As *EpiWATCH* sources global information, this methodology can be repeated for other diseases and other countries within the Western Pacific Region.
